# Parental Effect of Higher Education on Attitudes Towards Immigrants: A Family Approach

**DOI:** 10.1111/1468-4446.70005

**Published:** 2025-06-22

**Authors:** Victoria Donnaloja, Magda Borkowska

**Affiliations:** ^1^ University of Essex Wivenhoe Park Colchester UK

## Abstract

People with higher education hold more positive attitudes towards immigrants than those without. Previous studies have attempted to net out selection mechanisms to examine whether there is a causal effect of higher education on attitudes towards immigrants. However, parental higher education has been largely neglected as a likely source of this selection. Using UKHLS data on individuals and their parents for the UK and employing the khb decomposition model, we examine if and why parental education influences attitudes towards immigrants. First, we show that, net of individual educational attainment, individuals whose parents have a university degree are more likely to have more positive attitudes towards immigrants. More highly educated people have more positive attitudes, but parental education reinforces this association or compensates for low educational attainment. Second, we illustrate that the relationship between parental higher education and attitudes towards immigrants is mediated by two mechanisms: parental socialisation and individual education. In contrast, socio‐economic positioning while growing up makes a negligible contribution. Our findings suggest that formative years are crucial for the development of attitudes towards immigrants later in life and that educational inequalities of today affect the attitudes towards immigrants of tomorrow.

## 1 Introduction

One of the most robust findings in the literature on attitudes towards immigrants is that people with higher education hold more positive attitudes than the lower educated (Dražanová et al. 2024). Extant literature has devoted its efforts to identify whether this relationship is causal, that is whether educational attainment directly or indirectly affects attitudes towards immigrants (Finseraas and Kotsadam 2017; d’Hombres and Nunziata 2016; Cavaille and Marshall 2019; Margaryan et al. 2021). Scholars have therefore focussed entirely on individual educational attainment and, in doing so, they have largely overlooked the role of parental education, which is a likely source of endogeneity in the relationship. In this study we ask if and why people who have parents with a university degree have more positive attitudes towards immigrants than those whose parents do not.

There is disagreement on why education matters for attitude formation. According to one line of research, education enables the attainment of prestigious social positions which insulate individuals from competition by immigrants. Since higher qualifications are associated with a competitive advantage in the labour market, those at the top of the education distribution do not compete with immigrants who tend to be less educated and skilled (Scheve and Slaughter 2001; O'Rourke and Sinnott 2006; Mayda 2006; Borgonovi 2012). If this is the case, although the highly educated may be less vulnerable to competition than the low educated, they are nonetheless vulnerable to changes to increasing levels of immigration, globalisation and financial shocks. Moreover, over time, the strong positive association at the individual level between education and attitudes towards immigrants would not be replicated at the societal level because social position is a relational property. The competitive advantage of the highly educated should diminish as more people acquire higher education.

Alternatively, education may have an absolute liberalising effect on people's attitudes towards immigrants, by teaching relevant content and analytical skills, by cultivating tolerance as a core value and by teaching what attitudes are socially acceptable to hold (Stubager 2009). In this case, rising levels of education at the individual level would lead to rising levels of positive attitudes towards immigrants over time. This is so even if the effect of education is merely superficial on the attitudes people think they should have (Creighton et al. 2022; Janus 2010).

Finally, education may not matter at all. The association between education and attitudes may be spurious and therefore driven entirely by other factors, for example by personality traits (Lancee and Sarrasin 2015; Weber 2022; Velásquez and Eger 2022).

One important limitation of current literature is that it has not fully addressed the fact that new generations are closely connected to previous ones through family links (see Zagrean et al. 2022 for a review). This is an important underexplored aspect because at least part of the relationship between education and attitudes towards immigrants at the individual level may be rooted in the family through parental education. In other words, parental education is a likely source of endogeneity.

Parental education may influence individual attitudes towards immigrants through several channels. First, by focussing on individual education, the literature has not paid sufficient attention to the fact that people's social standing during adolescence may be more formative than socio‐economic status in adulthood. Parents' educational attainment is strongly associated with the social status of the family, including that of their children. Since attitudes towards immigrants form at an early age and are remarkably stable throughout the lifetime (Kustov et al. 2021; Devine and Valgarsson 2023), the effect of socio‐economic positioning on attitudes towards immigrants might happen before adulthood. Second, parents with higher levels of education may explicitly teach their children to have positive attitudes towards immigrants or, more indirectly, showcase behaviour that is consistent with their attitudes towards immigrants (Miklikowska 2016). In other words, the association between education and attitudes towards immigrants may be driven by parental education mediated by parental socialisation. Finally, parents' highest qualification is an important determinant of individual educational attainment (Bukodi and Goldthorpe 2013). People whose parents have a university degree are much more likely to have a degree themselves.

In this study we investigate the relationship between education and attitudes towards immigrants across generations by using the family as analytical site. Using ordered logistic regression, we investigate if higher parental education influences the formation of more positive attitudes towards immigrants in adult children. We also examine what mechanisms ‐socio‐economic, socialisation and/or individual educational attainment, mediate the relationship between parental education and one's attitudes towards immigrants using the KHB decomposition model. We show that socialisation rather than economic factors is the main channel through which parental education is associated with attitudes towards immigrants.

This paper makes several contributions. We shift the focus from the individual to the family to illustrate the inter‐generational effects of educational attainment on attitudes towards immigrants. We combine the literature on attitude formation with research on social mobility to conceptualise the relationship between educational inequalities and attitudes towards immigrants. The attitude formation literature contends that attitudes towards immigrants form early in life and shows that different generations are tightly linked to each other. The social mobility literature, has examined the effects of parental educational inequalities on the educational trajectories of their children. We build on this work to conceptualise and show that the relationship between educational inequalities and attitudes towards immigrants extends beyond the individual. In doing so, we move on from assessing that parental education influences political attitudes. We enrich the current theories of why education influences attitudes towards immigrants, by extending the role of socio‐economic privilege and socialisation for attitude formation from adulthood only to include adolescence.

## 2 Why Might Education Affect Attitudes Towards Immigrants?

One theory suggests that education has an absolute positive effect on attitudes towards immigrants. In democratic societies, education can counter feelings of hostility towards outsiders, including immigrants, by promoting the values of tolerance and equality (Hout 2012). University students may learn these values from their teachers and peers (Stubager 2008). This may be especially relevant in countries like the UK, where the student body is more diverse than broader society (Census 2021). Graduates are thus more likely to be inclusive and embrace cultural differences. Education also provides the tools to critically evaluate political discourse on immigration (Dame Adjin‐Tettey 2022), though it could also just teach to endorse socially acceptable views (Creighton et al. 2022; Janus 2010). Educational attainment may also make people more resilient to critical life events, as Kratz (2021) finds for Germany. If education drives positive attitudes towards immigrants, an increase in higher education participation should lead to a more inclusive society. The strongest evidence for a direct link between education and attitudes towards immigrants comes from research into the effects of school reforms in Western Europe that extended compulsory schooling between the 1940 and 1990s. Most of these studies, except for Norway (Finseraas and Kotsadam 2017), find that additional schooling led to more favourable views of immigration (d’Hombres and Nunziata 2016; Cavaille and Marshall 2019; Margaryan et al. 2021). Longitudinal studies usually find small, positive effects of higher education on liberal attitudes more generally once endogeneity is accounted for (Simon 2022; Scott 2022).

The positive effect of education may be overstated if it is relative to the educational attainment in society. According to the relative education model, education gives a competitive advantage that shields the highly educated from direct competition with immigrants in both the labour market and over public resources (Scheve and Slaughter 2001; O'Rourke and Sinnott 2006; Mayda 2006; Borgonovi 2012). More recent evidence shows that, rather than direct competition with the foreign‐born, what drives negative attitudes towards immigrants is having low transferable skills in the labour market (Pardos‐Prado and Xena 2019). Economic competition theories thus suggest that the more positive attitudes towards immigrants of the highly educated are the result of their privileged position in society. This would imply that as more people attain higher levels of higher education and therefore lose their competitive advantage, attitudes towards immigrants may not become more positive (Campbell 2006).

Longitudinal analyses have examined whether attitudes towards immigrants change with transitions within the educational system. Studies for Switzerland and Germany have found no individual changes in attitudes towards immigrants as people progress through the educational system. This suggests that there may be no effect of education on attitudes towards immigrants, and that the observed relationship is spurious (Weber 2022; Lancee and Sarrasin 2015). For example, personality traits that lead to more positive attitudes towards immigrants may also increase the likelihood of pursuing higher education. These traits could then largely explain the correlation between education and attitudes towards immigrants. We argue that parental educational attainment is also a likely source of endogeneity in this relationship that has not been properly examined.

## 3 From Individual to Family: The Role of Parental Educational Attainment

The level of education of one generation may very well affect the attitudes towards immigrants of the next because generations are tightly connected. The formation of attitudes towards immigrants is part of the political socialisation process. This process ranges from formal political education‐such as lessons in schools, civics courses, or media content‐to more informal forms of influence, such as the modelling of norms, values, and behaviours by different socialisation agents. These include family members, peers, religious leaders, and other figures in one's immediate social environment (Niemi and Sobieszek 1977). While it is acknowledged that political views, attitudes and affiliations can evolve throughout a person's life (Kinder 2006), there is widespread consensus that early life stages—particularly childhood and young adulthood—are crucial periods for cultivating political interest and attitudes, including attitudes towards immigrants (Krosnick and Alwin 1989). Recent evidence suggests that attitudes towards immigrants form at an early age and are stable thereafter (Kustov et al. 2021; Devine and Valgarsson 2023).

Due to the heightened sensitivity of early life, parents play a central role as agents of political socialisation, and much research focuses on how political interest, affiliation, and behaviours are passed from parent to child (Neundorf et al. 2017). Thus, parents are likely to influence the formation of attitudes towards immigrants.

Parental educational attainment is likely to play a role in the process of attitude formation (Persson 2015). Children of highly educated parents are found to be more politically engaged and more liberal (Paterson 2008; Surridge 2016). Margaryan et al. (2021) rely on the staggered implementation of a compulsory schooling reform in West Germany to identify the effect of mothers' educational attainment on their offspring’ concern for immigration. They find that the adult children of more educated mothers are less likely to be worried about immigration. Our starting point is therefore that.

**H1:** *Having a highly educated parent increases the likelihood of positive attitudes towards immigrants, net of individual higher education.*

To understand why parental education affects individual attitudes towards immigrants, we apply the theoretical underpinnings of the models of individual education to parental educational attainment. An absolute effect of parental education would work through parental socialisation. Formal education equips individuals with critical thinking skills, knowledge, and an interest in current events, meaning that highly educated parents are likely to teach their children to have positive attitudes towards immigrants or/and the values that underpin them. Moreover, highly educated parents are more likely to create a home environment that fosters positive attitudes towards immigrants by exhibiting positive behaviour that is consistent with their views, such as having a diverse friendship group. Adolescents typically take notice and emulate the behaviour of their parents, increasing the likelihood of holding similar attitudes towards immigrants (Miklikowska 2017). We therefore expect that parental socialisation mediates the relationship between parental education and attitudes towards immigrants. We test the following hypothesis.

**H2a:** *The relationship between parental educational attainment and attitudes towards immigrants is mediated by parental socialisation.*

Parental education may also affect attitudes towards immigrants indirectly through socio‐economic positioning. Parents with higher levels of educational attainment are also more likely to provide their children with a more privileged upbringing. In other words, educational attainment reflects social status: higher levels of education correlate with a more privileged social position. In turn, education can provide time, financial resources, and motivation necessary for political involvement (Kasara and Suryanarayan 2015). People with higher levels of education are more likely to be in higher earning occupations (Psacharopoulos and Patrinos 2018) and to be wealthier (Buckner and Abdelaziz 2023). Children from relatively less affluent backgrounds may hold less positive attitudes towards immigrants because they grow up feeling threatened by immigrants and immigration. If their parents experience direct competition with immigrants in the labour market or over public resources, they are exposed to a threatening environment despite not experiencing competition themselves. Financial stability while growing up is also associated with higher self‐efficacy, which in turn predicts more positive attitudes towards immigrants (Chen and Guo 2024; Crocetti et al. 2021). Better‐off adolescents have greater access to resources, such as books and computers, and cross‐cultural experiences, such as holidays abroad, that foster learning about other cultures. In sum, we expect that socio‐economic status during formative years mediates the relationship between parental education and attitudes towards immigrants. Therefore, we formulate the following hypothesis.

**H2b:** *The relationship between parental educational attainment and attitudes towards immigrants is mediated by socio‐economic positioning.*

Finally, it is well established that people whose parents hold a degree are more likely to have a degree themselves. They are in fact more likely to perform better academically early on because their parents are highly motivated to avoid downward mobility (Goldthorpe 2016). They also have more stable trajectories through higher education (Breen and Goldthorpe 1997; Haas and Hadjar 2020) and are more capable of foregoing the income they would earn if they entered the labour market earlier instead of staying in higher education. In the UK the effect of parental education on children's educational performance has even strengthened over time (Bukodi and Goldthorpe 2013). We expect that part of the relationship between parental educational attainment and attitudes towards immigrants is mediated by individual educational attainment. Therefore, we test the following hypothesis.

**H3:** *The relationship between parental educational attainment and attitudes towards immigrants is mediated by individual educational attainment.*

## 4 Analytical Approach

The analysis proceeds in two steps. To test H1 we run an ordered logistic regression (Model 1) on attitudes towards immigrants to account for the ordinal nature of the dependent variable. The dependent variable which captures respondents' attitudes, is measured on a scale ranging from strongly disagree to strongly agree. This approach respects the ordering of categories while avoiding the assumption of equal intervals between response options. Our model includes educational attainment, parental educational attainment, the interaction between the two, and it controls for level‐1 and level‐2 confounders. We do not include the parental variables that mediate the relationship between parental education and attitudes towards immigrants because in this first step we want to examine the overall association between parental education and attitudes towards immigrants, net of individual factors.

To test H2a‐H3 we apply the KHB method to decompose the total effect of parental educational attainment into a direct effect and indirect effects. The indirect effects are the ones that operate through other mediating variables. The KHB method is used in nonlinear probability models (Karlson and Holm 2011; Kohler et al. 2011). It compares the regression coefficients for the variable of interest (parental higher education) across two models: one that includes parental educational attainment and the mediators (Z); and one that includes parental education and a version of Z that has been residualised with respect to parental educational attainment. These two models have the same predictive power, but in the second model the residualised Z variables are uncorrelated with parental educational attainment. By comparing the coefficients of parental educational attainment between these models, the method isolates the effect of mediation on the coefficient for parental educational attainment. We use the Stata command KHB (Kohler and Karlson 2024) and we repeat these steps using an ordered logistic specification for the two outcomes under consideration. The output includes estimates of direct, indirect and total effects, as well as the percentage contribution of each mediator to indirect and total effects.

We calculate the marginal predicted probability of the interaction term between higher education and parental educational attainment on holding a positive attitude, based on Model 1 and present these in graphical form. Full results are in Table SM2 in the Supplementary Material (SM). To interpret the results of Model 2, we graphically present the proportion of the association between parental educational attainment and attitudes towards immigrants that is mediated by the variables included in the KHB model. This allows us to compare the extent to which each indirect path contributes to explaining the relationship between parental educational attainment and attitudes towards immigrants. Full results are in Tables SM3—SM5 in the SM.

## 5 Data and Measures

We utilize 12 waves of Understanding Society—the UK Household Longitudinal Study (UKHLS), which is uniquely positioned to address our research questions as it includes information on different family members, for example parents and their children. This allows us to link parental and children's data on both attitudes and socio‐economic factors. Our outcome variable, attitudes towards immigrants, is measured in Wave 12 (2020–2022) for both parents and children, as this is the only wave that includes questions on attitudes towards immigrants. We use the Special Licence version of the UKHLS data with Census 2011 Lower Layer Super Output Areas (LSOA) identifiers to link respondents' addresses to neighbourhood‐level indicators of diversity and deprivation (ONS 2011). Incorporating neighbourhood‐level characteristics enables us to account for the influence of the local environment on adolescents' attitude formation. This is important since highly educated people are more likely to live in more diverse and less deprived areas (Maxwell 2019). This therefore allows us to account for potentially important contextual factors that might affect attitudes, and to separate them from that of the family.

To address H1‐H2b, we select the sample comprising of UK‐born respondents aged 18–30 with at least one parent who are also survey respondents in wave 12. We limit the sample to this age group to allow us to link information about the children's parents from when they were aged 14 by accessing previous waves of the survey. We link parental information to one randomly selected sibling and assign it to all siblings in the group, ensuring they share the same parental data. We define siblings as biological and adopted siblings. These individuals are the most likely to share not only parents, but also other environmental characteristics that may confound the association between parental education and attitudes.

We select one parent per respondent. If the respondent has more than one parent, we select the one with the highest level of education or the mother if parents have the same level of educational attainment. This allows us to separate the effect of individual education from the effect of parental education. If we selected the parent with the lowest level of education we would be omitting the family‐level human capital that the individual had access to when growing up. This would therefore introduce bias. However, one may be concerned that for respondents with both parents, the less educated parent may have more influence than the selected parent on their child's attitudes towards immigrants. This could be, for example, due to the differences in parent‐child relationship quality (Zagrean et al. 2022). As a robustness check, we run the analysis on the sample of respondents whose parents are both present (*N* = 1625 complete cases). Such approach allows us to control for the non‐selected parent's educational attainment and its influence on individual attitudes. We only include the second parent's education and not their attitudes because this suffices to control for all sources of endogeneity arising from their attitudes (see Directed Acyclic Graphs in Figures SM1 and SM2 demonstrating this in the SM).

There are two outcomes of interest that measure attitudes towards immigrants: immigrants are good for Britain's economy‐henceforth ATI (economy) and Britain's culture is harmed by immigrants‐henceforth ATI (culture). They are measured on a five‐point Likert scale (strongly agree to strongly disagree). In the analysis for Model 1 we collapse the two categories that represent a positive view into one, that is for ATI (economy): strongly agree and agree are classified as a positive attitude. In the analysis for Model 2 we reverse code ATI (culture) for ease of interpretation.

Parental educational attainment is our independent variable of interest, which we measure as holding a university degree or not. We categorise respondents currently in higher education together with graduates. This is consistent with our focus on the selection process into positive attitudes towards immigrants, rather than on the effect of individual higher education. We do not categorise respondents with other higher education qualifications as graduates.

We include other independent variables hypothesised to mediate the relationship between parental educational attainment and attitudes towards immigrants. These include: individual educational attainment, parental socio‐economic class, wealth, income, and parental attitudes towards immigrants. Individual educational attainment is measured in the same way as the parental‐level variable; Parental socio‐economic status during adolescence is measured by occupational class using a four‐category NSSEC classification (management and professional, intermediate, routine, inactive (including students)). Housing tenure is used as a proxy of wealth; it differentiates between housing that is owned outright, owned with a mortgage, or rented. Income is captured by equivalised household income deciles, which adjust for inflation and household composition. These adjustments ensure that our income measure is comparable across time and across households of different sizes. All socio‐economic measures are observed when the respondent was around age 14 and living with their parents. Parental socialisation mechanism is measured by parental attitudes towards immigrants ‐ ATI (economy) and ATI (culture). These are entered‐separately for each respective outcome. Parental attitudes are observed in wave 12 when the respondent is an adult, rather than during adolescence.

In addition to the hypothesised mediators, we control for several factors. At both the individual and parental level, we control for sex (female/male), and age at wave 12. We also control for individual socio‐economic positioning at wave 12 (NSSEC), housing tenure, and equivalised household income deciles. Other control variables are included only at the parental level to avoid multicollinearity since many socio‐demographic characteristics correlate very highly between parents and their children and are unlikely to change over time. These are Government Office Region, and ethnicity (white/non‐white). We do not include voting preferences because these are likely to be a collider in the relationship between parental education and attitudes towards immigrants. At the neighbourhood level (defined as Lower Super Output Area ‐ LSOA), we include: an indicator of diversity measured as the proportion of non‐White respondents based on 2011 census (0‐1 scale); and Index of Multiple Deprivation deciles measured between 2010 and 2012.^1^ We choose to include geographical indicators as close as possible to 2011 as the 2011 census is closest to the time when our respondents were adolescents living in parental households.

The final sample has 3221 cases. However, note that specific sample size vary by Model specification and outcome (ATI (economy) or ATI (culture)) due to missingness. See Table 1 for a breakdown of sample characteristics, including parental characteristics and their missingness. No variable has a level of missingness above 10%. Table SM1 in the SM also compares our analytical sample to 18–30 in w12 to show they do not differ on any key characteristics.

**TABLE 1:** Respondent sample characteristics.

	Respondent	Parent
Variable	Frequency (proportion)	Frequency (proportion)
*N*	3221	
Sex		
Male	1424 (44.2%)	783 (24.3%)
Female	1797 (55.8%)	2438 (75.7%)
Age w12	23.278	53.643
Ethnicity		
Not white	713 (22.1%)	660 (20.5%)
White	2508 (77.9%)	2561 (79.5%)
NS‐SEC		
Management and professional	613 (19.0%)	1162 (36.1%)
Intermediate	348 (10.8%)	577 (17.9%)
Routine	586 (18.2%)	645 (20%)
Inactive	1369 (42.5%)	794 (24.7%)
Missing	305 (9.5%)	43 (1.3%)
Higher education		
Not highly educated	1515 (47.0%)	2101 (65.2%)
Highly educated	1697 (52.7%)	1094 (34%)
Missing	9 (0.3%)	26 (0.8%)
Housing tenure		
Owned outright	732 (22.7%)	520 (16.1%)
Owned with mortgage	1375 (42.7%)	1967 (61.1%)
Rent	1009 (31.3%)	721 (22.4%)
Missing	105 (3.3%)	13 (0.4%)
Income deciles	5.713	4.860
Missing	98 (3%)	26 (0.81%)
Immigrants are good for Britain's economy		
Strongly agree	924 (28.7%)	600 (18.6%)
Somewhat agree	1060 (32.9%)	990 (30.7%)
Neither agree nor disagree	924 (28.7%)	1098 (34.1%)
Somewhat disagree	184 (5.7%)	373 (11.6%)
Strongly disagree	124 (3.8%)	160 (5.0%)
Missing	5 (0.2%)	
Britain's culture is harmed by immigrants		
Strongly agree	85 (2.6%)	135 (4.2%)
Somewhat agree	260 (8.1%)	397 (12.3%)
Neither agree nor disagree	862 (26.8%)	1097 (34.1%)
Somewhat disagree	778 (24.2%)	800 (24.8%)
Strongly disagree	1229 (38.2%)	786 (24.4%)
Missing	7 (0.2%)	6 (0.2%)
Deprivation index LSOA11	—	4.523
Diversity index LSOA11	—	0.533

*Note:* Characteristics of respondents, where *p*_ stands for parent. Total sample size is 3221. See data section for full descriptions of variables.

In all the analysis, we cluster standard errors by siblingship, whereby standard errors are averaged between clusters. Even though we control for key parental and household characteristics, this deals with correlations of observations between siblings due to having grown up in the same household. Parental characteristics are treated as attributes of the respondents. We use wave 12 cross‐sectional weights provided with the survey data.

## 6 Findings

**FIGURE 1:**
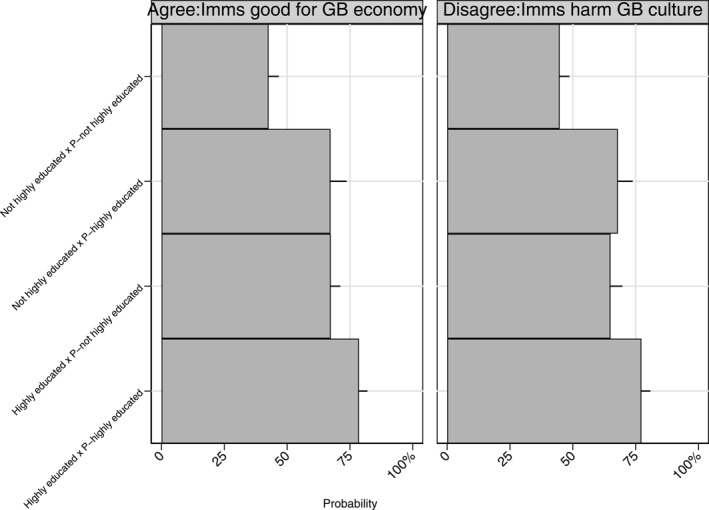
Predicted probability of holding a positive ATI (economy) and ATI (culture) by educational attainment. Predicted probability of agreeing and strongly agreeing with ATI (economy) and disagreeing and strongly disagreeing with ATI (culture) calculated from the ologit in Model 1; sample size are 2776 for ATI (economy) and 2772 for ATI (culture). P‐ refers to parent. Black lines delineate 95% confidence intervals.

Figure 1 illustrates the predicted probability of holding a positive view calculated from Model 1, among highly versus. non highly educated respondents who have a highly educated parent or not. For both ATI (economy) and ATI (culture), our results confirm H1: there is a clear association between parental educational attainment and attitudes towards immigrants. Among non‐highly educated and highly educated individuals alike, having a parent who is highly educated increases the probability of holding positive attitudes towards immigrants. There is a clear monotonic increase in the likelihood of holding positive attitudes associated with educational attainment. The probability goes from 43% ATI (economy) and 45% ATI (culture) for people who are not in higher education or graduates and whose parents are not either, to 67% ATI (economy) and 65% ATI (culture) for those who are in or have completed higher education, but their parents are not, to 67% ATI (economy) and 68% ATI (culture) for individuals who are not in higher education or graduates, but who have parents who are; to 79% ATI (economy) and 77% ATI (culture) for those who are in or have completed higher education with parents who have too. This means that having a highly educated parent compensates for not having a degree yourself. The probability of holding positive attitudes towards immigrants is in fact similar for those who are not highly educated, but who have a highly educated parent and for those who are highly educated, but do not have a highly educated parent.

**FIGURE 2:**
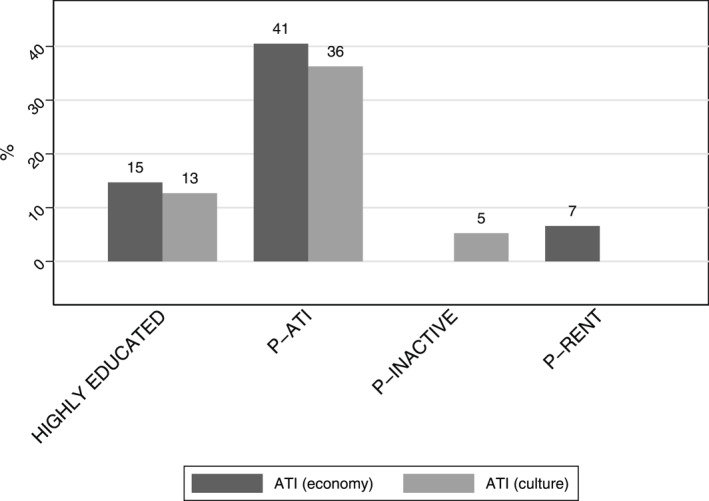
Contributors of mediators to total effect of parental educational attainment. The figure shows how much of the total effect of parental educational attainment is due to confounding of the statistically significant mediators estimated with the khb method separately for each outcome (Model 2); the sample sizes are 2706 ATI (economy) 2, 700 ATI (culture); ATI (culture) coding is reversed for interpretability. P‐RENT refers to parents renting the property rather than owning it; P‐inactive refers to parents who were not in work as opposed to managerial positions. The overall confounding percentage is 61% for ATI (economy) and 54% for ATI (culture). See Tables SM3—SM5 for full results.

We now move on to Model 2 to test if the association between parental education and attitudes towards immigrants is mediated by the mechanisms hypothesised in H2a‐H3. For full results of the KHB analysis see Tables SM3 and SM4 in the SM. The log odds of holding a negative attitude decrease by 1.004 for ATI (economy) and 0.926 for ATI (culture) for highly educated parents compared to lower educated ones (see Table SM3). This corresponds to a decrease in odds of 63% and 60%. Figure 2 illustrates the proportion of this total association between parental educational attainment and attitudes towards immigrants that is mediated by these mechanisms. We only include statistically significant mediators. Figure 2 shows that most of the positive association between parental education and attitudes towards immigrants is mediated by parental attitudes towards immigrants, therefore confirming H2a. 40% of the association between parental education and ATI (economy) is mediated by parental ATI (economy); and 38% of the association between parental education and ATI (culture) is mediated by parental ATI (culture). This suggests that parental socialisation is the main mechanism through which parental education affects attitudes towards immigrants. Parents who are highly educated are more likely to have children with positive attitudes towards immigrants because they themselves are more likely to have positive attitudes towards immigrants. It is plausible that they instil these attitudes in their children and model behaviours that align with those attitudes.

A smaller, yet statistically significant mediation path is the one through housing for ATI (economy). Renting as opposed to owning housing while growing up accounts for 7% of the association between parental higher education and ATI (economy). Similarly, having parents who were out of work as opposed to employed in management positions while growing up accounts for 4% of the association between parental higher education and ATI (culture). At face value these are counterintuitive findings. However, they are explained by the fact that the mediators here act as suppressors. In other words, the association between higher education and renting is negative, as is the association between renting and ATI (economy). Similarly, the association between higher education and economic inactivity is negative, as is the association between economic inactivity and ATI (culture). The indirect effects have a positive sign because they are the product of these two negative associations. The KHB command does not report these parameters in the output, but a simple investigation of these associations confirms this. See Tables SM6a to SM7b in the SM. It means that part of the indirect effect of parental education on attitudes towards immigrants operates through its influence on living in rented accommodation: parental education is negatively associated with living in rented housing, and living in rented housing is, in turn, negatively associated with attitudes towards immigrants. As a result, the product of these two negative relationships yields a positive indirect effect. Similarly, part of the positive effect of parental education on attitudes is due to the fact that more educated parents are less likely to be inactive, and being inactive is associated with less positive attitudes.

Finally, we find support for H3. For both outcomes we find that parental education is a source of endogeneity in the relationship between higher education and attitudes towards immigrants since higher education mediates part of that relationship. Although statistically significant, the contribution of this path is relatively small (14% for ATI (economy) and 13% for ATI (culture)). We should note that the direct effect of parental education remains statistically significant, net of the indirect effects discussed. This means that there are unobservable characteristics, such as personality traits, that children inherit from their highly educated parents and that lead to more positive attitudes towards immigrants.

The results of our robustness check, where we re‐run the models controlling for the educational attainment of the second parent for those with both parents in the sample, are robust to this specification. The only difference is that the effect of housing on ATI (economy) is no longer statistically significant. See Tables SM8 and SM9, and Figure SM3 in the SM for full results.

## 7 Discussion and Conclusion

Overall, we find that parental higher education influences attitude formation with respect to immigrants. Our evidence suggests that individuals with highly educated parents have more positive attitudes towards immigrants than those with low educated parents. This is primarily due to the fact that highly educated parents hold more positive attitudes towards immigrants than low educated parents.

By shifting the analytical lens from individual to family, we demonstrate that educational inequalities have long‐lasting effects on societal attitudes towards immigrants through inter‐generational transmission. It is true that individuals who are more educated have more positive attitudes towards immigrants than those who are less educated, but parental educational attainment has the power to reinforce this association or to compensate for low educational attainment. Although individuals can make up for the low educational attainment of their parents if they attend university, their likelihood of holding positive attitudes towards immigrants is still lower than those whose parents went to university. Individuals who have not attended university and whose parents have not attended university are the least likely to hold positive attitudes towards immigrants.

Through the decomposition model, our analysis contributes to unpacking the mechanisms through which parental education affects individual attitudes. We extend both the relative and absolute models of educational attainment to the period of adolescence. We find the strongest support for the socialisation mechanism—children of highly educated parents have positive attitudes towards immigrants largely because their parents have positive attitudes towards immigrants. It is therefore likely that the positive attitudes are transmitted through parental modelling during childhood and adolescence. We find weak evidence that better socio‐economic positioning during childhood mediates the relationship between parental higher education and attitudes towards immigrants. Part of the reason why the children of highly educated parents hold more positive attitudes towards immigrants is that they did not grow up in rented housing and did not have parents who were economically inactive. However, this contribution is small. Finally, we find that higher parental education contributes in small part to more positive individual attitudes by increasing the likelihood of individual higher education. This suggests parental education contributes to the selection process into higher education and more positive attitudes towards immigrants.

This study suffers from a few limitations. The sample is derivative and may therefore suffer from selection issues. We have selected respondents in wave 12 with at least one parent present in wave 12, who responded to the attitudinal questions of the survey. Therefore, parental attitudes towards immigrants are measured in wave 12, not while the child was growing up. The high correlation between parental and individual attitudes towards immigrants could be due to circumstance, such as political events, rather than socialisation. Moreover, many of the young individuals we include in the sample live with their parents at time of interview. It follows that the similarity between parents and their adult children may in part be driven by co‐habitation. However, we should note that living together is not a confounder in the relationship between parental education and attitudes towards immigrants as it does not correlate with parental education. Another limitation of measuring attitudes in wave 12 is that parental attitudes may have been different while the children were growing up. Recent evidence on graduates' lower propensity for euroscepticism shows that education takes time to shape attitudes (McNeil and Simon 2025). It is possible that the similarity in attitudes between parent and child we observe developed after adolescence. However, a large body of literature suggests that attitudes towards immigrants are stable in adulthood (Kustov et al. 2021; Devine and Valgarsson 2023), which reduces this concern. Measuring attitudes towards immigrants in the same wave rather than at different waves also allows to control for period effects. Finally, although we model relationships between parental educational attainment and attitudes towards immigrants using a mediation model, the associations we find are correlational, not causal. A consequence of that is that socialisation may work in the opposite direction, with children influencing their parents' attitudes rather than the other way round. Psychological research shows that the parent‐child relationship is reciprocal (Miklikowska 2016), which might account for some of the association between parental and individual attitudes.

Data that measure attitudes towards immigrants for both individuals and their parents are rare, even more so repeatedly over time. However, longitudinal designs that follow adolescents could causally examine how different educational trajectories of families translate into different attitudes towards immigrants later in life. Richer data could also help elucidate how parents transmit their attitudes towards immigrants to their children, if they do so consciously and if they combine direct teaching with behaviour that is coherent with their attitudes towards immigrants.

Overall, our findings suggest that formative years are crucial for the development of attitudes towards immigrants later in life. Parental education is part of that story because parents with different levels of education socialise their kids differently. This means that educational inequalities today affect the attitudes towards immigrants of tomorrow.

## Conflicts of Interest

The authors declare no conflicts of interest.

## Supporting information

Supporting Information S1

## Data Availability

The data that support the findings of this study are openly available in UK Data Service at http://doi.org/10.5255/UKDA‐SN‐6614‐20, reference number SN: 6614.
