# Supplementary material for: Parental Effect of Higher Education on Attitudes Towards Immigrants: A Family Approach
Source: Br J Sociol. 2025 Jun 22;76(5):965–74. doi: 10.1111/1468-4446.70005 (PMC12668244; doi:10.1111/1468-4446.70005)
Supplement: Supplementary file 1 — Supporting Information S1 [file BJOS-76-965-s001.docx]

Appendix for *The enduring effect of higher education on attitudes towards immigrants: a family approach*

**Sample description**

| **Table SM1a: Respondent sample characteristics** |  |  |
| --- | --- | --- |
|  | Our analytic sample (respondents aged 18-30, who had at least one parent in Understanding Society data, and valid responses to attitudes to immigrants for themselves and their parents) | Sample of 18-30 year olds in wave 12 Understanding Society data |
| N | 3,221 | 4,458 |
| **Sex** |  |  |
| Male | 1,424 (44.2%) | 1,923 (43.1%) |
| Female | 1,797 (55.8%) | 2,535 (56.9%) |
| **Age** | 23.278 | 23.897 |
| **Ethnicity** |  |  |
| Not white | 713 (22.1%) | 1,022 (22.9%) |
| White | 2,508 (77.9%) | 3,436 (77.1%) |
| **NS-SEC** |  |  |
| Management and professional | 613 (19.0%) | 910 (20.4%) |
| Intermediate | 348 (10.8%) | 521 (11.7%) |
| Routine | 586 (18.2%) | 820 (18.4%) |
| Inactive | 1,369 (42.5%) | 1,755 (39.4%) |
| Missing | 305 (9.5%) | 452 (10.1%) |
| **Highest qualification** |  |  |
| GCSE or lower | 502 (15.6%) | 743 (16.7%) |
| a-level | 788 (24.5%) | 1,098 (24.6%) |
| Degree | 1,146 (35.6%) | 1,692 (38.0%) |
| In Higher Education | 776 (24.1%) | 891 (20.0%) |
| Missing | 9 (0.3%) | 34 (0.8%) |
| **Housing tenure** |  |  |
| Owned outright | 732 (22.7%) | 877 (19.7%) |
| Owned with mortgage | 1,375 (42.7%) | 1,794 (40.2%) |
| Rent | 1,009 (31.3%) | 1,523 (34.2%) |
| Missing | 105 (3.3%) | 264 (5.9%) |
| **Mean income decile** | 5.713 | 5.619 |
| **Living with parents (Yes)** | 2,278 (70.72%) | 2,679 (60.09%) |
| **Living with parents and in higher education** | 713 (31.38%) | 792 (29.7%) |
| **Living with parents and have a degree** | 600 (26.41) | 711 (26.7%) |
| **Not living with parents and in higher education** | 63 (6.7%) | 99 (5.6%) |
| **Not living with parents and have a degree** | 546 (58.09%) | 981 (55.8%) |
| **Mean age if living with parents** | 22.19 | 22.28 |
| **Mean age if not living with parents** | 25.90 | 26.33 |

| **Table SM1b Respondent sample characteristics** | | |  | |  | |  |
| --- | --- | --- | --- | --- | --- | --- | --- |
|  | Highly educated parents, not-highly educated children | Highly educated parents, highly educated children | | Not-highly educated parents, not-highly educated children | | Not-highly educated parents, highly educated children | |
| **N** | 338 | 757 | | 1,167 | | 925 | |
| **Sex, derived** |  |  | |  | |  | |
| Male | 149 (44.1%) | 331 (43.7%) | | 545 (46.7%) | | 385 (41.6%) | |
| Female | 189 (55.9%) | 426 (56.3%) | | 622 (53.3%) | | 540 (58.4%) | |
| **Age** | 22.4 | 23.2 | | 23.5 | | 23.4 | |
| **Ethnicity** |  |  | |  | |  | |
| Not white | 54 (16.0%) | 167 (22.1%) | | 211 (18.1%) | | 257 (27.8%) | |
| White | 284 (84.0%) | 590 (77.9%) | | 956 (81.9%) | | 668 (72.2%) | |
| **NS-SEC** |  |  | |  | |  | |
| Management and professional | 55 (16.3%) | 200 (26.4%) | | 131 (11.2%) | | 223 (24.1%) | |
| Intermediate | 41 (12.1%) | 63 (8.3%) | | 157 (13.5%) | | 84 (9.1%) | |
| Routine | 106 (31.4%) | 45 (5.9%) | | 353 (30.2%) | | 77 (8.3%) | |
| Inactive | 101 (29.9%) | 410 (54.2%) | | 364 (31.2%) | | 476 (51.5%) | |
| Missing | 35 (10.4%) | 39 (5.2%) | | 162 (13.9%) | | 65 (7.0%) | |
| **Housing tenure** |  |  | |  | |  | |
| Owned outright | 85 (25.1%) | 205 (27.1%) | | 228 (19.5%) | | 205 (22.2%) | |
| Owned with mortgage | 160 (47.3%) | 350 (46.2%) | | 449 (38.5%) | | 407 (44.0%) | |
| Rent | 85 (25.1%) | 175 (23.1%) | | 455 (39.0%) | | 284 (30.7%) | |
| Missing | 8 (2.4%) | 27 (3.6%) | | 35 (3.0%) | | 29 (3.1%) | |
| **Income decile** | 6.5 | 6.9 | | 5.0 | | 5.5 | |
| **Living at home with parent(s)** |  |  | |  | |  | |
| yes | 241 (71.3%) | 517 (68.3%) | | 840 (72.0%) | | 655 (70.8%) | |
| **Parental NS-SEC** |  |  | |  | |  | |
| Management and professional | 210 (64.8%) | 501 (67.1%) | | 230 (19.8%) | | 214 (23.4%) | |
| Intermediate | 48 (14.8%) | 89 (11.9%) | | 219 (18.9%) | | 189 (20.7%) | |
| Routine | 32 (9.9%) | 66 (8.8%) | | 352 (30.4%) | | 234 (25.6%) | |
| Inactive | 34 (10.5%) | 91 (12.2%) | | 358 (30.9%) | | 277 (30.3%) | |
| ‘Highly educated’ children category includes those still in higher education  Overall, among parents with higher education, there is 31% of children without higher education (and not currently in higher education), and 69% of children with higher education or currently in higher education. | | | | | | | |

**Table SM2: Model 1 odds ratios from ologit on ATI**

|  | ATI (economy) | ATI  (culture) |
| --- | --- | --- |
|  |  |  |
| P-No HE X No HE | 1.000 | 1.000 |
|  | (.) | (.) |
| P-No HE X HE | 0.346^***^ | 0.424^***^ |
|  | (0.044) | (0.057) |
| P-HE X No HE | 0.348^***^ | 0.369^***^ |
|  | (0.059) | (0.060) |
| P-HE X HE | 0.191^***^ | 0.227^***^ |
|  | (0.026) | (0.030) |
| Management and professional | 1.000 | 1.000 |
|  | (.) | (.) |
| Intermediate | 0.997 | 0.850 |
|  | (0.164) | (0.148) |
| Routine | 1.281 | 1.450^*^ |
|  | (0.192) | (0.244) |
| Inactive | 1.302 | 1.352^*^ |
|  | (0.182) | (0.193) |
| Male | 1.000 | 1.000 |
|  | (.) | (.) |
| Female | 0.939 | 0.733^**^ |
|  | (0.089) | (0.069) |
| Age w12 | 1.009 | 1.034^*^ |
|  | (0.017) | (0.017) |
| Owned outright | 1.000 | 1.000 |
|  | (.) | (.) |
| Owned with mortgage | 0.891 | 0.941 |
|  | (0.101) | (0.120) |
| Rent | 1.014 | 1.093 |
|  | (0.134) | (0.171) |
| Income deciles | 0.949^**^ | 0.973 |
|  | (0.019) | (0.020) |
| P-Male | 1.000 | 1.000 |
|  | (.) | (.) |
| P-Female | 0.728^**^ | 0.861 |
|  | (0.084) | (0.096) |
| P age w12 | 0.978^*^ | 0.990 |
|  | (0.008) | (0.009) |
| North East | 1.000 | 1.000 |
|  | (.) | (.) |
| North West | 0.805 | 1.344 |
|  | (0.175) | (0.374) |
| Yorkshire and the Humber | 1.343 | 1.354 |
|  | (0.319) | (0.360) |
| East Midlands | 0.829 | 1.295 |
|  | (0.213) | (0.364) |
| West Midlands | 0.690 | 0.952 |
|  | (0.157) | (0.256) |
| East of England | 0.898 | 1.257 |
|  | (0.194) | (0.336) |
| London | 0.586^*^ | 0.998 |
|  | (0.136) | (0.277) |
| South East | 0.817 | 1.322 |
|  | (0.178) | (0.327) |
| South West | 0.759 | 1.129 |
|  | (0.177) | (0.307) |
| Wales | 0.915 | 1.227 |
|  | (0.216) | (0.338) |
| Scotland | 0.525^**^ | 1.010 |
|  | (0.113) | (0.272) |
| Northern Ireland | 1.264 | 2.506^**^ |
|  | (0.316) | (0.744) |
| Not white | 1.000 | 1.000 |
|  | (.) | (.) |
| White | 1.263 | 1.516 |
|  | (0.200) | (0.333) |
| Deprivation LSOA11 | 0.991 | 1.024 |
|  | (0.020) | (0.024) |
| Diversity LSOA11 | 1.054 | 0.742 |
|  | (0.234) | (0.177) |
| / |  |  |
| cut1 | 0.034^***^ | 0.451 |
|  | (0.022) | (0.298) |
| cut2 | 0.174^**^ | 1.360 |
|  | (0.109) | (0.896) |
| cut3 | 1.266 | 8.264^**^ |
|  | (0.793) | (5.409) |
| cut4 | 3.291 | 35.914^***^ |
|  | (1.911) | (24.301) |
| Observations | 2776 | 2772 |

Notes: Odds ratios with standard errors in parentheses. Lower values of both ATI refer

to more positive views.

Sample sizes are 2,776 for ATI (economy) and 2,772 for ATI (culture).

Standard errors in parentheses;

* p<0.05, ** p<0.01, *** p<0.001.

**KHB mediation**

The KHB mediation model estimates the total effect of parental education (*reduced* in the output); its direct effect (*full*) and its indirect effect, which is the difference between the two. If also includes standards errors for statistical significance of the indirect effect. In a second step it then estimates how much each mediators, if there are more than one, contributes to the total effect of parental education. This is presented in Tables SM3 and SM4. We have presented these in graphical form in Figure 3 in the manuscript.

**Table SM3: KHB significance test of mediation from Model 2**

|  | ATI (economy) | ATI  (culture) |
| --- | --- | --- |
| P-No HE  P-HE | - | - |
| Reduced | -1.004^***^ | -0.926^***^ |
|  | (-9.76) | (-8.72) |
| Full | -0.392^***^ | -0.426^***^ |
|  | (-3.30) | (-3.46) |
| Diff | -0.613^***^ | -0.500^***^ |
|  | (-7.98) | (-6.54) |
| Observations | 2706 | 2700 |
| Conf.-Ratio | 2.565 | 2.175 |
| Conf.-Perc. | 61.01 | 54.02 |

Notes: *t* statistics in parentheses.

Where the reduced model is the estimated total effect of P-higher education on the log-odds of ATI, the full model is the direct effect, the difference is the indirect effect. Conf-ratio is how many times larger the direct effect is than the tot effect; Conf. percentage refers to how much of the total effect is due to mediators.

^*^ *p* < 0.05, ^**^ *p* < 0.01, ^***^ *p* < 0.001

**Table SM4: Model 2 KHB Components of difference ATI (economy)**

|  |  |  |  | |  |
| --- | --- | --- | --- | --- | --- |
|  | Coef | Std_Err | P_Diff | P_Reduced | |
| P-HE |  |  |  | |  |
| HE | **-.1476991** | .0283881 | 24.09915 | | 14.70398 |
| P-Management  P-Intermediate | .0054146 | .0133099 | -.8834679 | | -.5390439 |
| P-Routine | -.0037464 | .0251196 | .6112755 | | .3729669 |
| P-Inactive | -.0282722 | .0185224 | 4.612997 | | 2.814598 |
| P-income deciles  P-Owned outright | .0178539 | .0293965 | -2.913114 | | -1.777423 |
| P- Owned with mortgage | .0166849 | .0168081 | -2.722368 | | -1.66104 |
| P-rent | **-.0662413** | .0264798 | 10.80819 | | 6.594567 |
| P-ATI(economy) | **-.4068753** | .0498662 | 66.38734 | | 40.50593 |

All mediators are included in the same model. Empty rows are reference categories for categorical variables. The first column adds up to the total indirect effect; coefficients that are statistically significant at the 5% level are marked in bold. Calculations are our own. The third column expresses the contribution of each mediator to the indirect effect; the last column shows how much of the total effect is due to confounding of the respective mediator and sums up to the overall confounding percentage.

**Table SM5: Model 2 KHB Components of difference ATI (culture)**

|  |  |  |  |  |
| --- | --- | --- | --- | --- |
|  | Coef | Std_Err | P_Diff | P_Reduced |
| P-HE |  |  |  |  |
| HE | **-.1175958** | .0258451 | 23.49946 | 12.69555 |
| P-Management  P-Intermediate | .0164883 | .0151027 | -3.294901 | -1.780066 |
| P-Routine | -.0272771 | .0272428 | 5.450858 | 2.944819 |
| P-Inactive | **-.0486635** | .0217666 | 9.72454 | 5.25367 |
| P-income deciles  P-Owned outright | .037254 | .0332482 | -7.444558 | -4.021913 |
| P- Owned with mortgage | -.0080122 | .0163553 | 1.601098 | .864991 |
| P-rent | -.0166097 | .0258044 | 3.319149 | 1.793166 |
| P-ATI(culture) | **-.3360033** | .0515353 | 67.14436 | 36.27465 |

All mediators are included in the same model. Empty rows are reference categories for categorical variables. The first column adds up to the total indirect effect; coefficients that are statistically significant at the 5% level are marked in bold. Calculations are our own. The third column expresses the contribution of each mediator to the indirect effect; the last column shows how much of the total effect is due to confounding of the respective mediator and sums up to the overall confounding percentage.

Figures SM1 and SM2: DAG for second parent confounders


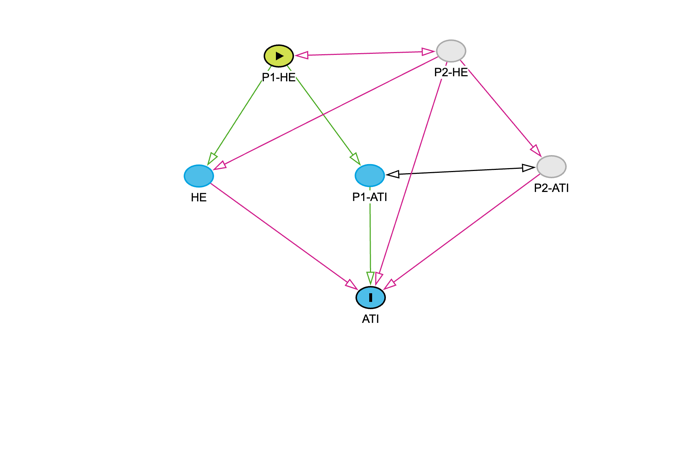

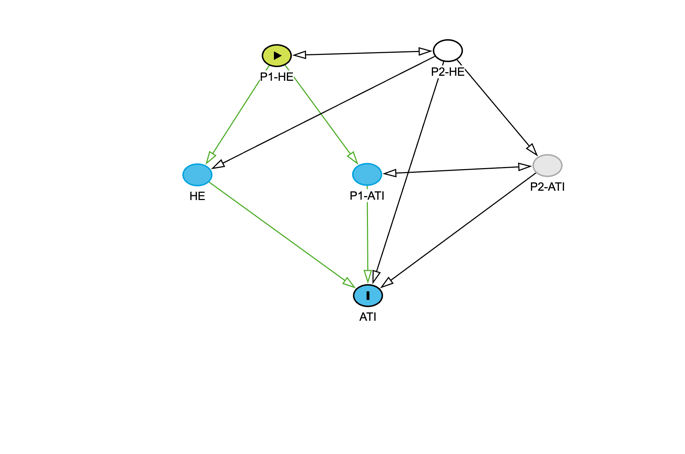
Notes: The DAG on the left shows in red the biasing paths that are open between P1-HE (highest educated parent's educational attainment) and ATI when P2-HE (lowest educated parent's educational attainment) and P2-ATI are unobserved. On the right, we show that by adjusting for P2-HE only all paths are closed off and any bias is accounted for.

**Table SM6a: cross-tab parental home ownership and parental higher education**

| P-home ownership | P-low educated | P-highly educated | Total |
| --- | --- | --- | --- |
| P-home owned outright | 291  13.90% | 222  20.39% | 513  16.12% |
| P-home owned with a mortgage | 1,174  56.06% | 773  70.98% | 1,947  61.17% |
| P-rented home | 629  30.04% | 94  8.63% | 723  22.71% |
| Total | 2,094  100% | 1,089  100% | 3,183  100% |

**Table SM6b: ATI (economy) and parental home ownership**

| Immigrants are good for GB economy | P-home owned outright | P-home owned with a mortgage | P-rented home | Total |
| --- | --- | --- | --- | --- |
| Strongly agree | 180  34.16% | 573  29.37% | 163  22.48% | 916  28.6% |
| Somewhat agree | 183  34.72% | 688  35.26% | 186  25.66% | 1,057  33% |
| Neither agree not disagree | 122  23.15% | 529  27.11% | 271  37.38% | 922  28.79% |
| Somewhat disagree | 24  4.55% | 107  5.48% | 53  7.31% | 184  5.74% |
| Strongly disagree | 18  3.42% | 54  2.77% | 52  7.17% | 124  3.87% |
| Total | 527  100% | 1,951  100% | 725  100% | 3,203  100% |

**Table SM7a: cross-tab parental NSSEC and parental higher education**

| P-nssec | P-low educated | P-highly educated | Total |
| --- | --- | --- | --- |
| P-management and professional | 435  20.90% | 715  67.20% | 1,150  36.57% |
| P-intermediate | 439  21.1% | 143  13.44% | 582  18.51% |
| P-routine | 561  26.96% | 90  8.46% | 651  20.7% |
| P-inactive | 646  31.04% | 116  10.9% | 762  24.23% |
| Total | 2,081  100% | 1,064  100% | 3,145  100% |

**Table SM7b: ATI (culture) and parental NSSEC**

| Immigrants harm GB culture | P- management and professional | P- intermediate | P-routine | P-inactive | Total |
| --- | --- | --- | --- | --- | --- |
| Strongly disagree | 500  43.37% | 220  37.93% | 213  32.77% | 277  35.47% | 1,210  38.24% |
| Somewhat disagree | 312  27.06% | 152  26.21% | 146  22.46% | 156  19.97% | 766  24.21% |
| Neither agree not disagree | 249  21.6% | 158  27.24% | 195  30% | 245  31.37% | 847  26.77% |
| Somewhat agree | 73  6.33% | 38  6.55% | 75  11.54% | 72  9.22% | 258  8.15% |
| Strongly agree | 19  1.65% | 12  2.07% | 21  3.23% | 31  3.97% | 83  2.62% |
| Total | 1,153  100% | 580  100% | 650  100% | 781  100% | 3,164  100% |

**Table SM8: Robust Model 1 odds ratios from ologit on ATI**

|  | ATI (economy) | ATI  (culture) |
| --- | --- | --- |
|  |  |  |
| P-No HE X No HE | 1.000 | 1.000 |
|  | (.) | (.) |
| P-No HE X HE | 0.342^***^ | 0.381^***^ |
|  | (0.065) | (0.073) |
| P-HE X No HE | 0.357^***^ | 0.374^***^ |
|  | (0.085) | (0.083) |
| P-HE X HE | 0.198^***^ | 0.226^***^ |
|  | (0.039) | (0.043) |
| P2-No HE | 1.000 | 1.000 |
|  | (.) | (.) |
| P2- HE | 0.675^*^ | 0.645^*^ |
|  | (0.129) | (0.125) |
| Management and professional | 1.000 | 1.000 |
|  | (.) | (.) |
| Intermediate | 0.859 | 0.808 |
|  | (0.201) | (0.188) |
| Routine | 1.434 | 1.457 |
|  | (0.280) | (0.299) |
| Inactive | 1.376 | 1.341 |
|  | (0.262) | (0.251) |
| Male | 1.000 | 1.000 |
|  | (.) | (.) |
| Female | 0.906 | 0.753^*^ |
|  | (0.110) | (0.093) |
| Age w12 | 1.008 | 1.039 |
|  | (0.023) | (0.023) |
| Owned outright | 1.000 | 1.000 |
|  | (.) | (.) |
| Owned with mortgage | 0.870 | 0.789 |
|  | (0.127) | (0.128) |
| Rent | 0.897 | 0.901 |
|  | (0.177) | (0.185) |
| Income deciles | 0.969 | 0.990 |
|  | (0.026) | (0.028) |
| Male | 1.000 | 1.000 |
|  | (.) | (.) |
| Female | 0.708^*^ | 0.816 |
|  | (0.111) | (0.123) |
| P age w12 | 0.987 | 0.976^*^ |
|  | (0.012) | (0.012) |
| North East | 1.000 | 1.000 |
|  | (.) | (.) |
| North West | 0.726 | 0.910 |
|  | (0.207) | (0.278) |
| Yorkshire and the Humber | 1.518 | 1.106 |
|  | (0.462) | (0.342) |
| East Midlands | 0.869 | 0.998 |
|  | (0.266) | (0.352) |
| West Midlands | 0.875 | 0.780 |
|  | (0.277) | (0.254) |
| East of England | 0.961 | 0.892 |
|  | (0.264) | (0.296) |
| London | 0.438^**^ | 0.701 |
|  | (0.137) | (0.256) |
| South East | 0.989 | 1.156 |
|  | (0.257) | (0.333) |
| South West | 0.891 | 1.141 |
|  | (0.269) | (0.380) |
| Wales | 1.127 | 1.233 |
|  | (0.332) | (0.402) |
| Scotland | 0.640 | 0.902 |
|  | (0.181) | (0.301) |
| Northern Ireland | 1.479 | 1.980 |
|  | (0.463) | (0.716) |
| Not white | 1.000 | 1.000 |
|  | (.) | (.) |
| White | 1.016 | 1.694^*^ |
|  | (0.238) | (0.378) |
| Deprivation LSOA11 | 1.007 | 1.009 |
|  | (0.027) | (0.028) |
| Diversity LSOA11 | 0.941 | 0.904 |
|  | (0.271) | (0.282) |
| / |  |  |
| cut1 | 0.047^***^ | 0.176^*^ |
|  | (0.041) | (0.149) |
| cut2 | 0.256 | 0.534 |
|  | (0.219) | (0.452) |
| cut3 | 1.829 | 3.371 |
|  | (1.564) | (2.841) |
| cut4 | 5.198 | 18.795^***^ |
|  | (4.422) | (16.633) |
| Observations | 1625 | 1622 |

Notes: Odds ratios with standard errors in parentheses. Lower values of both ATI refer

to more positive views.

Standard errors in parentheses;

* p<0.05, ** p<0.01, *** p<0.001.

**Table SM9: KHB significance test of mediation from Model 2 (robust)**

|  | ATI (economy) | ATI (culture) |
| --- | --- | --- |
| P-No HE  P HE | - | - |
| Reduced | -0.986^***^ | -0.897^***^ |
|  | (-6.22) | (-5.85) |
| Full | -0.453^**^ | -0.354^*^ |
|  | (-2.66) | (-2.08) |
| Diff | -0.533^***^ | -0.543^***^ |
|  | (-5.29) | (-5.22) |
| Observations | 1585 | 1580 |
| Conf.-Ratio | 2.176 | 2.532 |
| Conf.-Perc. | 54.05 | 60.50 |

*t* statistics in parentheses.

Where the reduced model is the estimated total effect of P-higher education on the log-odds of ATI, the full model is the direct effect, the difference is the indirect effect. Conf-ratio is how many times larger the direct effect is than the tot effect; Conf. percentage refers to how much of the total effect is due to mediators

^*^ *p* < 0.05, ^**^ *p* < 0.01, ^***^ *p* < 0.001
